# Announcement

**Published:** 2015-04-24

## National Campaign to Prevent Falls in Construction — United States, 2015

In 2013 and 2014, construction employment began to recover from the 2007–2009 economic downturn. In 2014, construction employment grew to 9.8 million workers from 8.9 million workers in 2012 (1). In 2013, there were 796 fatal work-related injuries in the private construction sector, accounting for the highest number of fatal work injuries of any industry sector (2,3). Falls on construction sites are the leading cause of death in the industry (36% of deaths in 2012) (4). Many construction occupations require working at height and climbing ladders or scaffolds on a daily basis; the falls occur mostly from roofs, scaffolds, and ladders (5). However, deaths and injuries from falls in construction are a preventable public health problem.

CDC’s National Institute for Occupational Safety and Health (NIOSH) continues its work with construction sector stakeholders through a government-labor-management partnership, representing state and federal government agencies, professional organizations, trade associations, labor organizations and private industry who worked together to develop a national campaign aimed at construction contractors, onsite supervisors and workers.

During May 4–15, the federal Occupational Safety and Health Administration (OSHA) and stakeholders including NIOSH, will host a National Safety Stand-Down to Prevent Falls in Construction (additional information available at http://www.osha.gov/StopFallsStandDown). The stand-down will be a voluntary opportunity for construction-related employers to speak directly to employees about fall hazards and to reinforce the importance of fall prevention requirements. It is part of a national information and media construction falls prevention campaign. In 2014, almost 5,000 local stand-downs were reported to OSHA, with participation in all 50 states. Broad engagement and promotion across the United States is encouraged, including by state agencies and public health practitioners.
